# Is Fibular Sesamoidectomy a Viable Option for Sesamoiditis? A Retrospective Study

**DOI:** 10.7759/cureus.4939

**Published:** 2019-06-19

**Authors:** Jeffrey M Pearson, Leonardo V M Moraes, Kyle D Paul, Jianguang Peng, Karthikeyan Chinnakkannu, Haley M McKissack, Ashish Shah

**Affiliations:** 1 Orthopaedic Surgery, University of Alabama School of Medicine, Birmingham, USA; 2 Orthopedics, Instituto De Assistência Médica Ao Servidor Público Estadual (IAMPSE), São Paulo, BRA; 3 Orthopaedics, University of Alabama School of Medicine, Birmingham, USA; 4 Orthopaedic Surgery, Xuanwu Hospital Capital Medical University, Beijing, CHN

**Keywords:** hallux, metatarsal, postoperative complications, sesamoid, sesamoidectomy

## Abstract

Background

Pathologic conditions of the sesamoids can be a source of disabling pain for patients, particularly during toe-off. Some underlying causes include osteonecrosis, inflammation, arthritis, and fracture. Nonoperative treatment is the initial standard of care, and has demonstrated satisfactory outcomes overall; however, operative management may be indicated in cases of pain refractory to conservative management. Sesamoidectomy is an uncommon procedure with risk of potential complications, but may be warranted in select cases of failed nonoperative treatment.

Methods

A retrospective chart review was conducted at one institution from 2009 to 2018. Twelve patients diagnosed with fibular sesamoiditis were treated with sesamoidectomy. Baseline patient demographics as well as postoperative outcomes were recorded.

Results

All 12 patients underwent fibular sesamoidectomy using the plantar approach following which their symptom (pain) resolved. Average follow-up for this cohort was 35 months. Of the sample, two patients experienced transient neuritis, one patient developed a superficial infection, and one had painful postoperative scarring. Hallux varus deformity was not observed in any patients.

Conclusion

Fibular sesamoidectomy may be a safe, viable procedure for patients with sesamoiditis who fail conservative measures.

## Introduction

The first metatarsophalangeal joint sesamoids play multiple roles in proper biomechanical functioning of the foot [1]. The sesamoids also increase the distance of the flexor tendons from the first metatarsophalangeal joint, which minimizes joint reaction forces. Additionally, they function as a pulley mechanism to increase the effective moment of the flexor hallucis brevis muscle, thereby generating strength [2]. Unfortunately, despite their biomechanical value, the sesamoids are often a source of pain and discomfort to patients. The sesamoids can be affected by a variety of pathologies including acute fracture, acute separation of bipartite sesamoids, sesamoiditis caused by repetitive trauma, infection, chondromalacia, osteochondritis dissecans, and osteoarthritis [3-6].

The treatment methods for sesamoid pathologies vary from conservative managements such as rest, ice, nonsteroidal anti-inflammatory medications, physiotherapy, and custom modified orthoses to surgery [3]. Conservative management is first line, and orthotics in particular play a key role in off-loading the hallux metatarsal phalangeal complex and stretching the gastrocnemius [7,8]. Surgery, if indicated, may involve tendo-Achilles or gastrocnemius lengthening; dorsiflexion osteotomy at the base of the first metatarsal; corrective osteotomies; fusions for the fixed pes cavus foot; or sesamoidectomy. Sesamoidectomy is a relatively uncommon procedure, but should be carefully considered if 6-12 months of conservative management fails, or if the patient experiences ongoing debilitating symptoms [5]. Many authors have noted the potential complications of sesamoidectomy including hallux varus, stiffness, painful scarring, wound dehiscence, wound infection, nerve injury, hallux valgus, and even cock up deformity [9-12]. Despite this, sesamoidectomy still remains a plausible treatment for cases of chronic sesamoidal pain in patients who have failed nonoperative treatment [13-15].

We present a series of patients who underwent fibular sesamoidectomy for intractable sesamoiditis that failed conservative measures. The purpose of this study was to evaluate the postoperative clinical outcomes and complications of this procedure. We hypothesize that fibular sesamoidectomy is a safe, effective treatment with few complications when properly performed in select cases.

## Materials and methods

After approval from our institutional review board, a retrospective chart review was performed from 2009 to 2018. Billing codes were reviewed, and all patients with a diagnosis of fibular sesamoiditis were identified with ICD-10 code M86.6. The cohort was further stratified based on operative intervention with fibular sesamoidectomy using the Current Procedural Terminology (CPT) code 28315.

A total of 12 patients met our criteria. We obtained demographic information including age, gender, body mass index (BMI), laterality, tobacco use, occupation, duration of conservative management, and comorbidities. In July 2018, all patients were contacted to complete the foot function index (FFI) via phone as well [15]. One patient who had rheumatoid arthritis (RA) was deceased, and four patients did not fill out the questionnaire.

According to preoperative images, two patients had hallux valgus and hallux rigidus. Of these two patients, one also had preexisting rheumatoid arthritis. Both the patients who had hallux valgus along with rigidus did not reply to our questionnaire. One patient in the cohort had isolated hallux rigidus with sesamoiditis. Seven of 12 patients completed the FFI for functional evaluation. Postoperative information collected included any evidence of infection, neuritis, return to operating room (OR), and postoperative deviation of the hallux. Any other postoperative complications, as well as total length follow-up, were recorded for each patient.

All patients were initially treated for an extended period with conservative measures. This included orthotics, anti-inflammatory medications, physical therapy, limitation of activity, and a trial of non-weight bearing. Despite these measures, symptoms persisted for these 12 patients. All patients underwent fibular sesamoidectomy for their symptoms.

**Figure 1:**
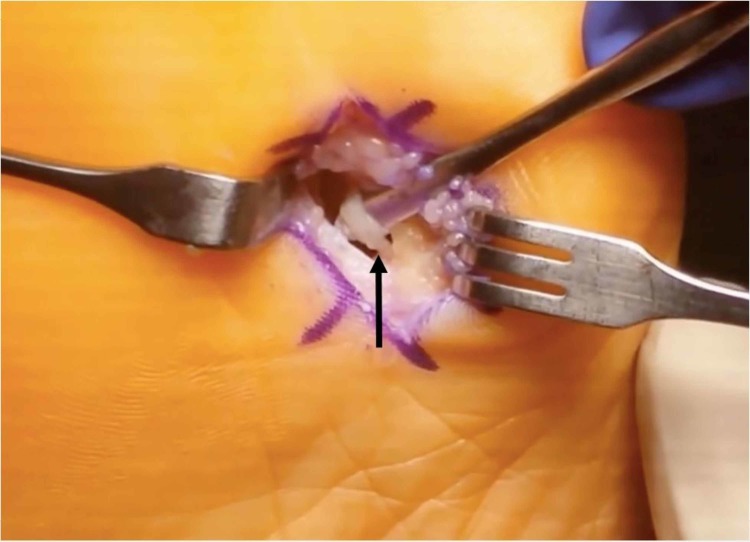
Plantar incision with isolated plantar digital nerve (black arrow)

**Figure 2:**
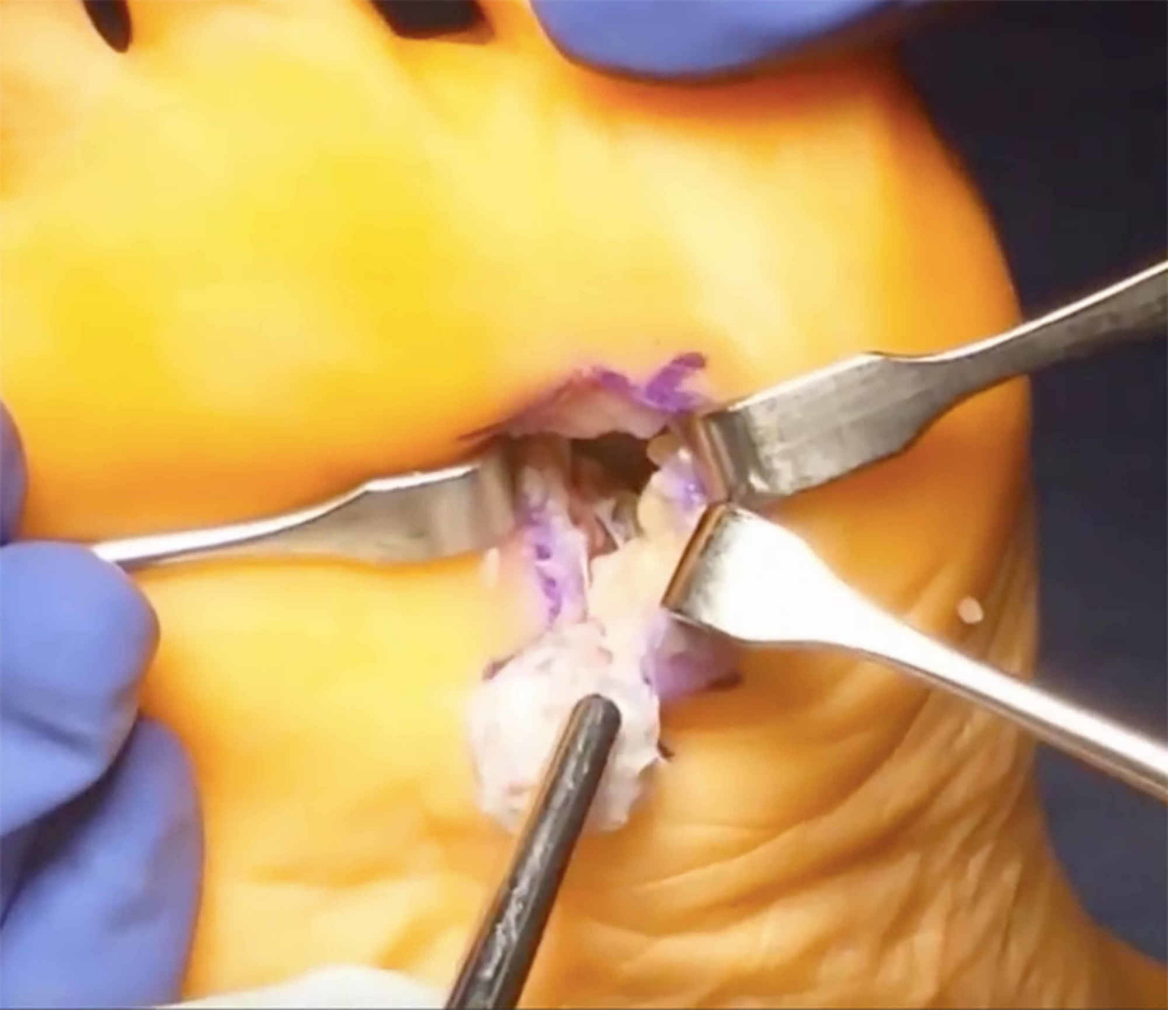
Lateral sesamoid excision

**Figure 3:**
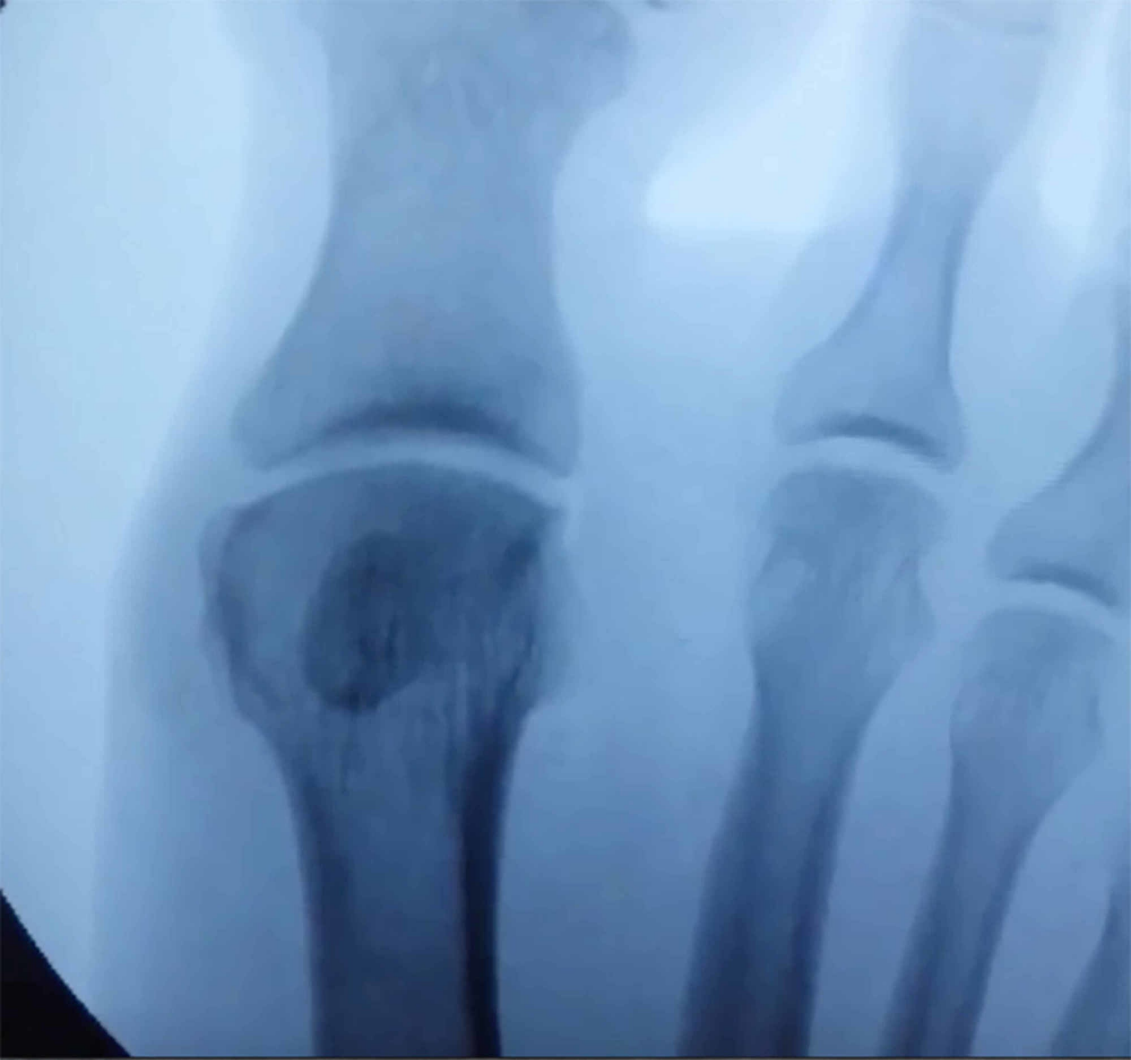
Intraoperative image after sesamoid excision

**Figure 4:**
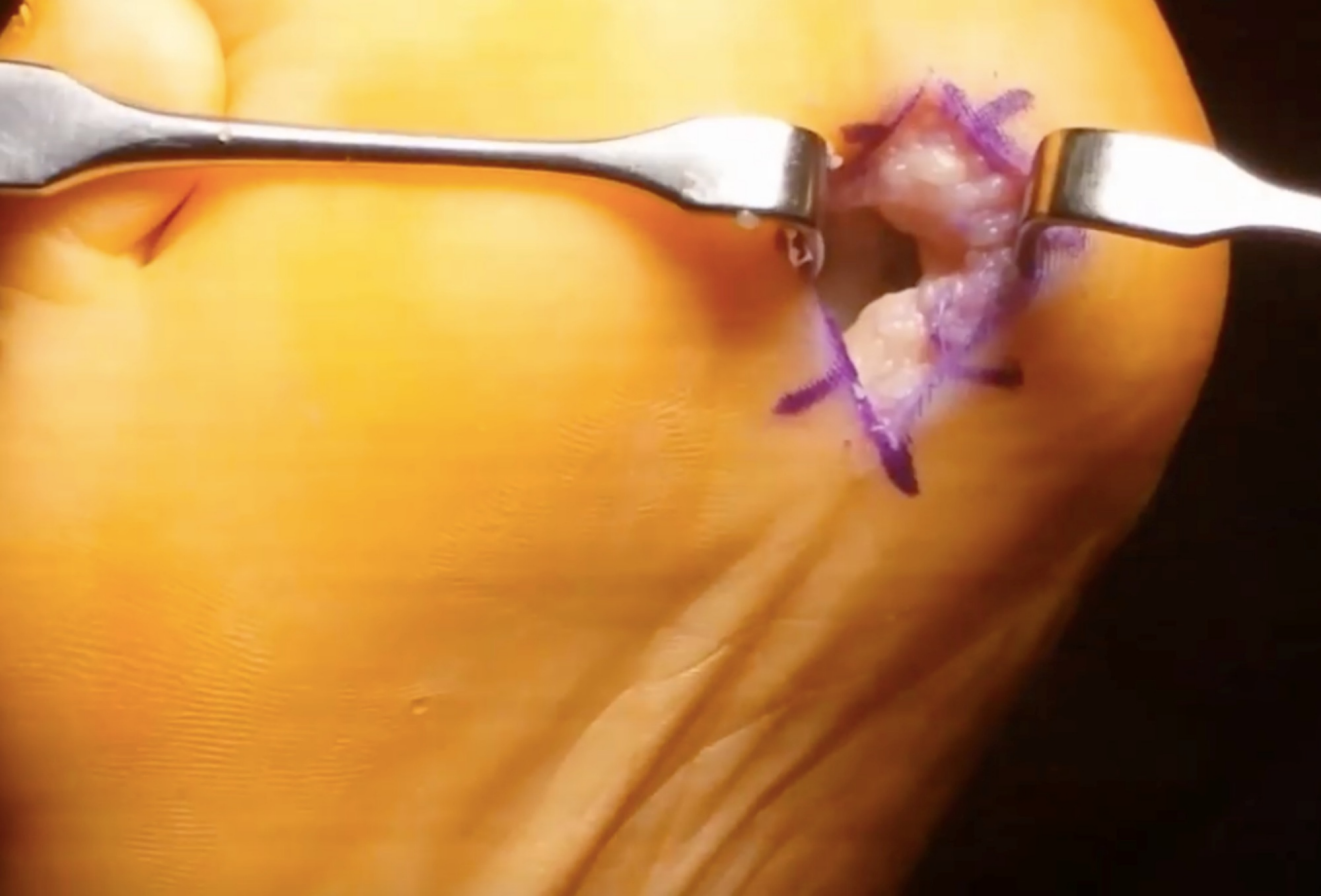
Plantar plate defect after sesamoid excision

**Figure 5:**
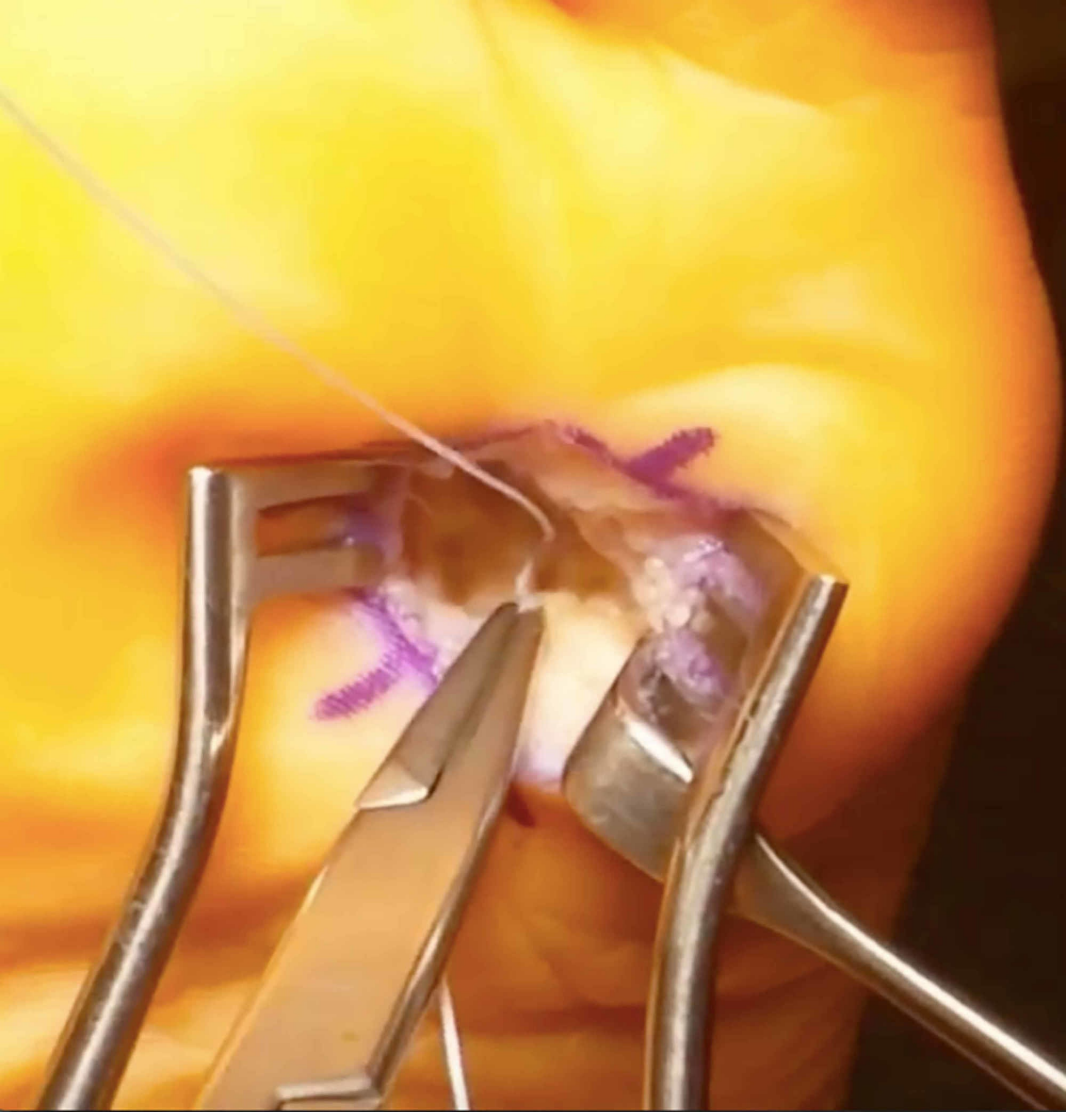
Plantar plate repair

The fibular sesamoidectomy procedure was performed by any one of three fellowship-trained foot and ankle surgeons. All surgeons used the plantar approach with a longitudinal incision on the lateral edge of the first metatarsal fat pad. Care was taken to ensure that the lateral plantar digital nerve was protected during the procedure (Figure 1). The metatarsal fat pad was retracted medially with the lateral plantar digital branch of the hallux, a branch of the medial plantar nerve. The lateral sesamoid was then isolated and shelled out with a beaver blade (Figure 2). The tendon of the flexor hallucis longus was inspected to ensure its continuity. The plantar plate was closed carefully to minimize postoperative great toe deviation (Figures 3-5). The skin was approximated with nonabsorbable sutures, avoiding placement of subcutaneous nonabsorbable suture material beneath the plantar skin. The foot was placed in bunion-type wound dressing with the hallux held in slight plantar flexion. Postoperatively, patients were kept non-weight bearing for two weeks, and were kept in a post-op walking shoe for six weeks total. Sutures were removed at two weeks postoperatively.

## Results

Preoperative patient baseline demographics can be found in Table 1. Of our 12 patients, the average age was 38 years. Ten of 12 patients (83%) were female, while two were male. The patients’ average BMI was 23.58. The majority of the patients (10) had no history of trauma, and two referred forefoot injury in the past.

**Table 1:** Baseline patient demographics BMI: Body mass index

Characteristic	Number of Patients
Average age (years)	37.58
Gender	
Female	10
Male	2
Average BMI	23.58
Side of surgery	
Right	8
Left	4
Tobacco use	1
History of trauma	2
Comorbidities	
Rheumatoid arthritis	1
Diabetes	1

Preoperative findings are shown in Table 2. According to preoperative images, two patients had both hallux valgus and hallux rigidus. One patient had preexisting rheumatoid arthritis with involvement of the first metatarsophalangeal (MTP) joint. Five of 12 (42%) patients had avascular necrosis of the sesamoid, based on magnetic resonance imaging (MRI).

**Table 2:** Preoperative findings AVN: Avascular necrosis; RA: Rheumatoid arthritis.

Finding	Number of Patients
Preoperative X-rays	
Hallux rigidus	3 (2 with concurrent hallux valgus)
Hallux valgus	2 (both with concurrent hallux rigidus; one with concurrent RA)
Preoperative exam findings	
Cavus foot	3
Previous sesamoid fracture	2
MRI findings	
AVN of sesamoid	5
Chronic changes	1

The average follow-up for the cohort was 35 months. No patients developed cock-up deformity of the lesser toes or hallux varus deformity either clinically or radiologically. Of the 12, two patients developed transient neuritis postoperatively, one developed a painful scar, and one developed a superficial infection. Among the seven patients who responded to the FFI questionnaire, the average score was 8.3. No patients required reoperation (Table 3).

**Table 3:** Patient characteristics and postoperative outcomes DM: Diabetes mellitus; RA: Rheumatoid arthritis; MTP: Metatarsophalangeal; BMI: Body mass index; FFI: Foot function index.

Patient Number	Gender	Age	BMI	Comorbidities	Other Foot Conditions	Smoking	Previous Trauma	Postoperative Complication	FFI
Isolated fibular sesamoiditis									
1	M	39	N/A	No		No	No	No	0
2	M	43	N/A	DM		No	No	No	0
3	F	31	N/A	No		No	No	No	3.9
4	F	30	18.88	No		No	No	Transient neuritis	N/A
5	F	36	30.41	No		No	No	Transient neuritis	10
6	F	43	22.67	No		No	Yes	Painful scar	N/A
7	F	44	21.84	No		No	Yes	No	N/A
8	F	21	25.6	No		No	No	No	8.7
9	F	29	24.7	No		No	No	No	21.7
Sesamoiditis with hallux rigidus									
10	F	59	N/A	RA	Hallux valgus, first MTP arthritis	Yes	No	No	N/A
11	F	33	23.57	No	Hallux valgus with rigidus	No	No	Superficial infection	N/A
12	F	43	20.98	No	Rigidus	No	No	No	14

## Discussion

Lateral sesamoidectomy is indicated when conservative management for sesamoiditis fails. Sesamoiditis is a broad term referring to conditions causing pain of the seasamoids, including avascular necrosis, overloading, chondromalacia, and fracture. In contrast to early studies, which report sesamoidectomy to be associated with high incidence of complications (particularly hallux varus), recent literature suggests improved outcomes [11,13,16,17]. However, most of these reports focus on combinations of medial, lateral, and paired excision, rather than lateral excision alone, making this one of the few reports in the literature that assesses operative outcomes of isolated fibular sesamoidectomy. Additionally, it includes one of the largest cohorts of isolated fibular sesamoidectomy for lateral insidious sesamoiditis to date, and is the first study to our knowledge which reports midterm follow-up results with a cohort of this size.

It is important to assess outcomes of isolated lateral sesamoidectomy, as this procedure has an increased likelihood of complications when compared to medial and paired excision. In a previous study by Kane et al., complication rates were found to be greater in lateral sesamoidectomy (41.2%) (seven of 17 patients) than in medial sesamoidectomy (22.2%) (four of 18 patients) [18]. Although these rates were not statistically significantly different, this may be due to small sample size, and does not necessarily reflect their clinical significance. Complications reported in the literature after sesamoidectomy include impaired range of motion, continued pain, neuroma development, transient neuritis, and deformities [19,20]. One patient in our study developed a superficial infection, which was successfully treated with oral antibiotics. No cases of neuroma occurred in our cohort, and two instances of neuritis were found which resolved with pregabalin. The results of our study yielded a lower incidence of neurological complications compared to other studies assessing operative outcomes in lateral sesamoidectomy.

Incidence of deformity was found to be lower in the current study than in previous reports documenting outcomes of lateral sesamoidectomy [16]. Hallux varus deformity is a potential consequence of weakness and instability at the first MTP joint, which may occur if the integrity of the flexor hallucis brevis (FHB), adductor hallucis tendons, or capsular structures is not maintained or restored [5]. Mann and Coughlin reported an 8% incidence of hallux varus after sesamoidectomy using the plantar approach [16]. In our study, after an average of 35 months postoperative follow-up, no patients developed cock up deformity, hallux varus, or hallux valgus. These results are comparable to outcomes reported by Milia et al. [13]. We believe that repairing the plantar plate and maintaining the continuity of FHB is crucial in preventing the occurrence of hallux varus deformity. Three patients in this series underwent concurrent plantar plate repair during the lateral sesamoidectomy procedure. One patient had comorbid rheumatoid arthritis and underwent concurrent Keller resection of the metatarsal head. One had hallux rigidus and underwent cheilectomy. The third patient had hallux valgus and underwent correction of the deformity with distal metatarsal osteotomy.

Utilization of the plantar approach for sesamoidectomy has been debated [1,13]. Mann et al. discouraged the use of this approach due to risk of development of a painful scar postoperatively [1]. Similarly, one patient from our cohort complained of a painful scar on the plantar aspect of the foot. However, Milia et al. reported no cases of painful scar formation or wound healing complications in a case of 12 patients treated with fibular sesamoidectomy through a plantar approach [13]. The direct plantar approach allows for direct visualization of the sesamoid and surrounding soft tissue structures, minimizes the violation of normal anatomic structures, and enables repair of the flexor hallucis brevis tendon following sesamoidectomy [13].

There are several limitations of this study, including those inherent to a retrospective chart review. The small number of patients in our cohort makes it difficult to come to any definitive conclusion. Considering the uncommon nature of the procedure, it was difficult to have powered study. Furthermore, only seven of 12 (58.33%) patients completed the post-operative follow-up questionnaires. It would have been beneficial to have a higher number of patient outcomes scores completed. Additionally, we did not have preoperative Foot Function Index evaluations for patients in the study. Despite these limitations, this initial retrospective review is a way to further research in the field for assessment of fibular sesamoidectomy as an appropriate procedure in select patients.

## Conclusions

In summary, fibular sesamoidectomy can be safely performed on patients who have failed conservative measures for sesamoiditis. The plantar lateral approach allows for adequate exposure of the fibular sesamoid, repair of the plantar plate, and preservation of FHB, and is beneficial in preventing the occurrence of hallux varus deformity.
